# Cranial midline shift is a predictor of the clinical prognosis of acute cerebral infarction patients undergoing emergency endovascular treatment

**DOI:** 10.1038/s41598-023-48401-0

**Published:** 2023-11-29

**Authors:** Xiao-Min Xu, Hao Zhang, Ren-Liang Meng

**Affiliations:** 1 Department of Neurology, The Affiliated Hospital of Southwest Medical University, Taiping Street, Jiangyang District, Luzhou, 646000 Sichuan China; 2 Laboratory of Neurological Diseases and Brain Function, Luzhou, Sichuan China

**Keywords:** Biomarkers, Neurology, Risk factors

## Abstract

Endovascular treatment is widely used in acute cerebral infarction (ACI), but patient prognosis varies greatly. We aimed to investigate the predictive value of midline shift (MLS) threshold for the clinical prognosis of patients with ACI who undergo emergency endovascular treatment. We prospectively enrolled patients with ACI who received endovascular treatment within 24 h of onset. Cranial images were collected within 24 h after endovascular treatment. We assessed MLS at the level of the midbrain, pineal calcification, septum pellucida, and falx cerebri and noted the maximum MLS (MLS[max]) among these locations. Functional outcomes were assessed at 90 days using the modified Rankin Scale. Receiver operating characteristic curves and optimal cutoff points were used to analyze the predictive value of MLS. We enrolled 82 patients, including 46 with poor outcomes. Although the MLS values at all levels were significantly different between the poor and favorable outcome groups (*p* < 0.01), the MLS(max) tended to be a better marker for 90-day poor outcome. To predict poor outcome, the optimal cutoff values for MLS(max) within 24 and 48 h after intervention were 0.45 and 2.35 mm, respectively. MLS(max) has predictive value for patient prognosis.

## Introduction

Stroke is a public health problem worldwide, second only to cardiovascular disease and cancer^1,2^. With an annual incidence of more than 13 million cases^3,4^, it is the second leading cause of death after myocardial infarction, and the third leading cause of disability^1,5^.

Ischemic stroke accounts for 71% of all strokes worldwide, of which 20% are caused by large vessel occlusion^5^. In the treatment of acute cerebral infarction (ACI), rapid recovery of the effective reperfusion of brain tissue is the primary aim. Current methods of recanalization include intravenous thrombolysis and endovascular therapy. However, intravenous thrombolysis has a relatively short time window and there are many restrictive conditions for its use. Patients who can be treated with recombinant tissue plasminogen activator account for just 2.4–5.2% of all patients with ACI^6^, and the recanalization rate of great vessels is only 13–18%^7^. In recent years, endovascular treatments such as mechanical thrombectomy have been widely used in patients with ACI. These have achieved good therapeutic effects in vascular recanalization, especially of large vessels, which is important for the rehabilitation and improved prognosis of patients. However, because of the risk of complications such as brain tissue reperfusion injury, cerebral vascular rupture and hemorrhage, and secondary cerebral edema, individual patient prognosis varies greatly. Assessing disease progression at the first critical juncture is crucial because early prediction and timely intervention may improve patient outcomes.

Calculation of the midline shift (MLS) threshold is a simple method for quantifying the early mass effect. It has been reported that different MLS thresholds are associated with different outcomes in patients with intracerebral hemorrhage (ICH)^8–15^ and malignant infarct^16–18^. Studies have demonstrated that a maximum MLS value (MLS[max]) > 4 mm is associated with poor prognosis in patients with ICH^8^, that an MLS > 3.7 mm in follow-up computed tomography (CT) approximately 24 h after the onset of severe ACI can predict a malignant course^16^, and that patients with horizontal pineal displacement > 4 mm on CT within 48 h of ACI have a high risk of early death^17^. We therefore aimed to explore the relationship between MLS and clinical prognosis in patients with ACI who received endovascular treatment within 24 h of onset, and to further determine the optimal threshold of MLS for predicting poor prognosis.

## Methods

### Patient selection and data collection

We prospectively enrolled patients who received endovascular treatment within 24 h of ACI onset in the Affiliated Hospital of Southwest Medical University from January 2020 to December 2020. Criteria for endovascular treatment eligibility were as follows: > 18 years of age; had ACI within the previous 24 h; had one or more large or medium vessel occlusions at the internal carotid artery, M1–M3 middle cerebral artery segments, or A2–A3 anterior cerebral artery segments (as determined by CT angiography or digital subtraction angiography); had a National Institutes of Health Stroke Scale (NIHSS) score > 6 within 6 h of onset and no obvious core infarction in a baseline non-contrast CT, or CT perfusion imaging within 24 h of onset indicated a serious mismatch between infarction core and low-perfusion area, with a large ischemic penumbra; and informed consent was obtained from the patient or an acceptable patient surrogate. Patients were eligible for our study if they received cranial CT or magnetic resonance imaging within 24 h after intervention and agreed to complete a telephone follow-up 3 months later. Data collected included demographic characteristics, medical history, clinical characteristics, preoperative NIHSS score, biochemical results, imaging data, and prognosis.

The study was approved by the Ethics Committee of the Affiliated Hospital of Southwest Medical University, and written informed consent was obtained from all participants or their legal representatives prior to participation.

### Outcome measures

Functional outcomes were assessed using the modified Rankin Scale (mRS) at 3 months via a telephone interview conducted by trained medical staff. This approach was chosen to ensure maximum follow-up rates and limited attrition. We defined a favorable outcome as asymptomatic to mild disability (mRS 0–2) and a poor outcome as moderate disability to death (mRS 3–6).

### Imaging

Cranial CT or magnetic resonance imaging was performed within 24 h after endovascular treatment. If a patient needed to complete several imaging reexaminations because of their condition, we selected the last imaging session. If possible, cranial images from 24 to 48 h after endovascular treatment were also collected for subgroup analysis. The brain midline was determined by creating a line connecting the anterior and posterior insertions of the falx cerebri. The maximum vertical distances from the center of the midbrain, pineal calcification, septum pellucida, and falx cerebri to the midline of the brain were measured and recorded as the MLS^8^. If each level was present on multiple CT slices, the maximum value selected from all MLS values was recorded. The MLS(max) was defined as the maximum MLS among all MLS locations. If the patient’s brain midline was deviated, the MLS value after intervention was subtracted from the MLS value of the first preoperative image after admission or the most recent head image before admission. MLS values (in mm) were measured independently by two observers and corrected for magnification. The infarct volumetry study was performed using the final CT scan with 5-mm sections of the patient during hospitalization. Infarcted lesion volume was measured using the *ABC*/2 formula, where *A*, *B*, and *C* represent the dimensions of the maximum level of the infarcted lesion in three perpendicular axes^17^. If the final infarcts were sporadic, the volumes were calculated separately and then summed.

### Statistical analysis

Data analyses were performed using IBM SPSS Statistics, version 21.0 (Armonk, NY, USA). Clinical characteristics of patients with mRS > 2 were compared with those of patients with mRS ≤ 2 using Pearson’s chi-squared test for categorical data, the Mann–Whitney *U* test for non-normal data, or Student’s *t*-test for normal data (as appropriate). Categorical data are reported as absolute numbers and percentages (%), continuous data with normal distributions are shown as mean values ± standard deviations, and continuous data with non-normal distributions are provided as medians and interquartile ranges (IQRs). Multivariate logistic regression analysis was performed to investigate independent factors correlated with poor outcome.

Receiver operating characteristic curve analysis was conducted to determine whether MLS values were predictive of 90-day poor outcome. The optimal MLS value was calculated using the optimal cutoff, sensitivity, specificity, area under the curve, and Youden’s index. The intraclass correlation coefficient, equivalent to the quadratic weighted Kappa statistic, was calculated to assess interobserver variability in MLS at the levels of the midbrain, pineal calcification, septum pellucida, and falx cerebri, as well as to evaluate MLS(max) consistency. Tests were two-tailed and significance was chosen as *p* < 0.05. The distribution of 90-day mRS scores was divided into two groups according to the MLS threshold.

### Ethical approval

All procedures performed in studies involving human participants were in accordance with the ethical standards of the institutional and/or national research committee and with the 1964 Helsinki Declaration and its later amendments or comparable ethical standards.

## Results

In the present study, 82 patients were enrolled; 46 cases (56.1%) had poor outcomes (mRS > 2) and 36 cases (43.9%) had favorable outcomes (mRS ≤ 2). The mean age of patients was 62.2 years and 39% were female. Clinical characteristics are shown in Table 1. All patients with previous minor stroke sequelae were classified into the poor prognosis group (17.4% vs. 0.0%, *p* = 0.024), whereas the good prognosis group contained a higher proportion of patients with hypertension (72.2% vs. 50%, *p* = 0.042). In the poor prognosis group, the final cerebral infarction volume was significantly higher than that in the good prognosis group (77.88 [IQR 25.25–133.15] vs. 14.81 [IQR 2.03–64.52], *p* < 0.001), and a higher proportion of patients needed decompressive craniectomy (19.6% vs. 2.8%, *p* = 0.049).

**Table 1:** Baseline characteristics in patients with or without poor outcome.

Variables	mRS > 2 (n = 46, 56.1%)	mRS ≤ 2 (n = 36, 43.9%)	p
Sex (male)	25 (54.3)	25 (69.4)	0.164
Age (year)	57.5 [54.8–71.3]	60.0 [52.0–71.5]	0.581
Smoking	14 (30.4)	17 (47.2)	0.120
Alcohol consumption	13 (28.3)	14 (38.9)	0.309
Hypertension	23 (50)	26 (72.2)	0.042
Diabetes	11 (23.9)	10 (27.8)	0.691
Atrial fibrillation	20 (43.5)	10 (27.8)	0.143
Pre-stroke mRS score	0 [0-0]	0 [0-0]	0.009
Patients with sequelae of previous stroke	8 (17.4)	0 (0.0)	0.024
Baseline mRS score	4 [4-4]	4 [4-4]	0.917
Baseline NIHSS score	13.4 ± 3.9	12.6 ± 4.7	0.392
TOAST subtypes (large-artery atherosclerosis)	26 (56.5)	24 (66.7)	0.350
Intravenous thrombolytic therapy	8 (17.4)	13 (36.1)	0.054
Time between operation and onset (h)	10 [5.8–14.0]	9 [6.0–13.8]	0.940
Duration of operation (min)	137.5 [103.8–173.5]	126 [82.5–153.8]	0.174
anesthesia (general anesthesia)	28 (60.9)	20 (55.6)	0.628
Hemispheric infarct localization (right)	20 (43.5%)	14 (38.9%)	0.675
Large or medium vessel occlusions (large vessel)	36 (78.3%)	29 (80.6%)	0.799
Preoperative TICI-scores	0 [0–0]	0 [0–0]	0.783
Postoperative TICI-scores (≥ IIb)	41 (89.1)	35 (97.2)	0.332
Postoperative intracranial hemorrhage	21 (45.7%)	12 (33.3%)	0.259
hematoma growth	3 (6.5%)	0 (0.0%)	0.333
Decompression surgery required	9 (19.6)	1 (2.8)	0.049
Decompression surgery	4 (8.7%)	0 (0.0%)	0.194
Final infarction volume (ml)	77.88 [25.25–133.15]	14.81 [2.03–64.52]	< 0.001
Time of postoperative CT review (h)			
Within 24 h	16.1 ± 4.88	16.2 ± 4.43	0.881
Within 48 h	2 5 [17.75–38.0]	25 [18.25–38.0]	0.877
MLS location within 24 h			
Midbrain shift (mm)	0 [0–2.2]	0 [0–0]	< 0.001
Pineal gland shift (mm)	1.1 [0–2.65]	0 [0–0]	0.001
Septum pellucidum shift (mm)	1.6 [0–4.2]	0 [0–0]	< 0.001
Cerebral falx shift (mm)	0 [0–2.725]	0 [0–0]	0.002
MLS(max) (mm)	2.55 [0–4.2]	0 [0–0]	< 0.001
MLS location within 48 h			
Midbrain shift (mm)	0 [0–2.3]	0 [0–0]	0.003
Pineal gland shift (mm)	1.8 [0–3.425]	0 [0–0]	0.001
Septum pellucidum shift (mm)	2.85 [0–4.375]	0 [0–0]	< 0.001
Cerebral falx shift (mm)	0 [0–3.4]	0 [0–0]	0.001
MLS(max) (mm)	3.0 [0–5.0]	0 [0–1.6]	< 0.001

NIHSS, National Institute of Health stroke scale; CT, computed tomography; MLS, midline shift; MLS(max), maximal MLS for all MLS locations; ICA, internal carotid artery; MCA, middle cerebral artery; ACA, anterior cerebral artery; Large vessel, lumen diameters upper 2.0 mm: ICA, M1 segment of MCA; Medium vessel, lumen diameters between 0.75 and 2.0 mm: M2-3 segment of MCA and A2-A3 segment of ACA.

The MLS values at the levels of the midbrain, pineal calcification, septum pellucida, and falx cerebri were significantly different between the poor and favorable outcome groups within both 24 and 48 h after intervention (*p* < 0.01). The MLS(max) values of patients with poor outcomes were higher than those of patients with good outcomes (within 24 h, 2.55 [IQR 0–4.2] vs. 0 [IQR 0–0], *p* < 0.001; within 48 h, 3.0 [IQR 0–5.0] vs. 0 [IQR 0–1.6], *p* < 0.001). In the multivariate logistic regression model, after adjusting for hypertension, pre-stroke mRS score, and final infarction volume, 90-day poor outcomes were associated with MLS values at the levels of the midbrain (adjusted odds ratio [OR] 3.919, 95% confidence interval [CI] 1.179–13.032, *p* = 0.026) and septum pellucidum (adjusted OR 2.071, 95% CI 1.177–3.643, *p* = 0.012), MLS(max) within 24 h (adjusted OR 2.237, 95% CI 1.336–3.748, *p* = 0.002), and MLS(max) within 48 h (adjusted OR 1.487, 95% CI 1.023–2.161, *p* = 0.037) (Table 2).

**Table 2:** Multivariate analysis of predictors for poor outcome.

Variables	Odds ratio	95% confidence interval	p
MLS location within 24 h			
Midbrain shift^a^	3.919	1.179–13.032	0.026
Pineal gland shift^a^	1.709	0.952–3.066	0.073
Septum pellucidum shift^a^	2.071	1.177–3.643	0.012
Cerebral falx shift^a^	1.735	0.871–3.460	0.117
MLS (max)^a^	2.237	1.336–3.748	0.002
MLS location within 48 h			
Midbrain shift^a^	1.193	0.791–1.798	0.400
Pineal gland shift^a^	1.300	0.846–1.998	0.231
Septum pellucidum shift ^a^	1.411	0.959–2.076	0.081
Cerebral falx shift^a^	1.665	0.947–2.927	0.077
MLS (max)^a^	1.487	1.023–2.161	0.037

MLS, midline shift; MLS(max), maximal MLS for all MLS locations.

^a^Adjusted for hypertension, pre-stroke mRS score, final infarction volume.

**Figure 1:**
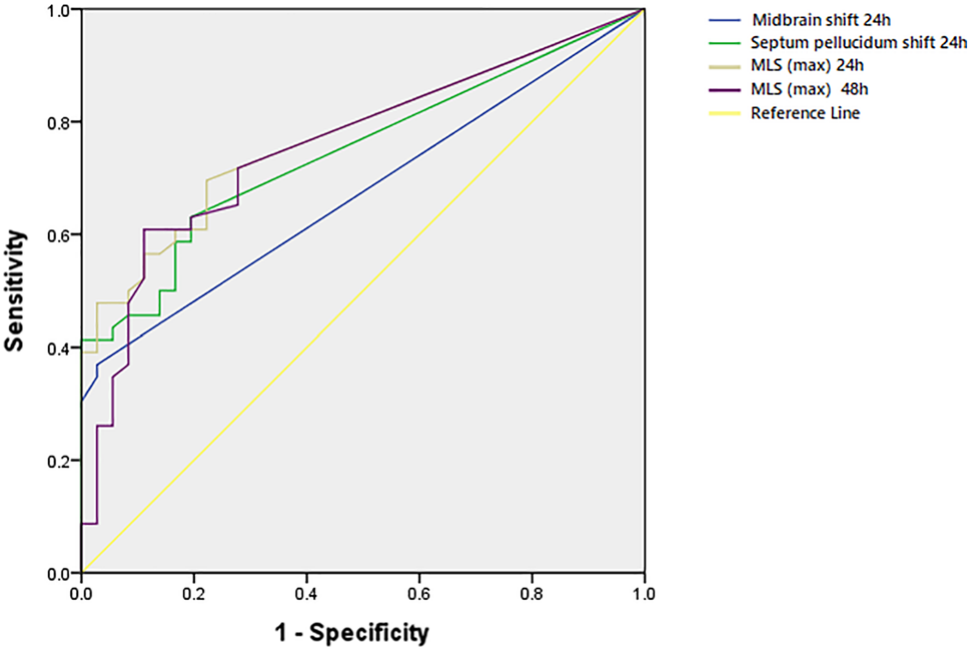
ROC curves for predicting 90-day poor outcome, a comparison of AUC among all MLS locations.

The receiver operating characteristic curves of MLS values for predicting 90-day poor outcomes are presented in Fig. 1. The MLS(max) tended to be a better neuroimaging marker for 90-day poor outcomes. The area under the curve of MLS(max) was 0.777 within 24 h and 0.758 within 48 h after intervention. To predict poor outcome at 90 days, the optimal cutoff values for MLS(max) within 24 and 48 h after intervention were 0.45 mm (Youden’s index = 0.473) and 2.35 mm (Youden’s index = 0.498), respectively (Table 3).

**Table 3:** MLS locations associated with 90-day poor outcome.

Variables	AUC	YI	Optimal cutoff	Sensitivity	Specificity
MLS location within 24 h					
Midbrain shift	0.675	0.342	0.7	0.370	0.972
Septum pellucidum shift	0.748	0.436	0.5	0.630	0.806
MLS (max)	0.777	0.473	0.45	0.696	0.778
MLS location within 48 h					
MLS (max)	0.758	0.498	2.35	0.609	0.889

MLS, midline shift; MLS(max), maximal MLS for all MLS locations; AUC, area under the curve; YI, Youden's index.

**Figure 2:**
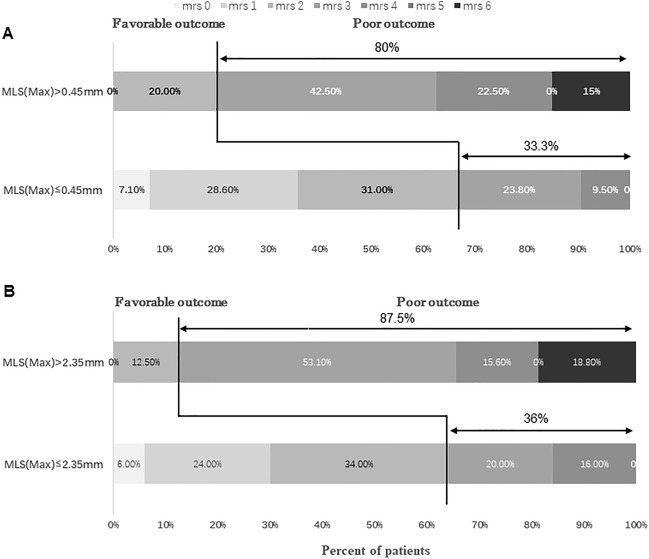
Distribution of 90-day mRS in patients with and without MLS(max) threshold; (**A**) within 24 h after intervention; (**B**) within 48 h after intervention.

We divided patients into two groups using the optimal thresholds of MLS(max). As shown in Fig. 2, the prognosis of the MLS(max) > 0.45 mm group was worse than that of the MLS(max) ≤ 0.45 mm group within 24 h after intervention, (mRS > 2, 80% vs. 33.3%, respectively, *p* < 0.001; death rates, 15% vs. 0%, respectively, *p* = 0.029) and the prognosis of the MLS(max) > 2.35 mm group was worse than that of MLS(max) ≤ 2.35 mm group within 48 h after intervention (mRS > 2, 87.5% vs. 36%, respectively, *p* < 0.001; death rates, 18.8% vs. 0%, respectively, *p* = 0.006). When using values from within 24 h after intervention, 25% of patients with MLS(max) > 0.45 mm required decompression craniotomy compared with 0% of patients with MLS(max) ≤ 0.45 mm (*p* = 0.002). Furthermore, the 90-day mRS scores of 85.7% of patients in the MLS(max) ≤ 0.45 mm group were improved by a median of 2 points [IQR 1.0–3.0], whereas those of 57.5% (*p* = 0.004) of patients in the MLS(max) > 0.45 mm group were improved by a median of 1 point[IQR 0–1.0] (*p* < 0.001). When using values from within 48 h after intervention, 31.3% of patients with MLS(max) > 2.35 mm required decompression craniotomy compared with 0% of patients with MLS(max) ≤ 2.35 mm (*p* < 0.001). Moreover, the 90-day mRS scores of 82.0% of patients in the MLS(max) ≤ 2.35 mm group were improved by a median of 2 points[IQR 1.0–3.0], whereas those of 56.3% (*p* = 0.011) of patients in the MLS(max) > 2.35 mm group were improved by a median of 1 point [IQR − 0.75 to 1.0] (*p* < 0.001).

The intraclass correlation coefficient between the two observers for the MLS(max) within 24 h after intervention was 0.974 (95% CI 0.96–0.983); for the MLS(max) within 48 h after intervention it was 0.975 (95% CI 0.962–0.984).

## Discussion

In patients with ICH^8–15^ and malignant infarct^16–19^, MLS on CT has been widely investigated. In the present study, we aimed to investigate the predictive value of MLS for the clinical prognosis of patients with ACI who undergo emergency endovascular treatment. In this prospective study, we enrolled patients with ACI who received endovascular treatment within 24 h of onset, and demonstrated for the first time that MLS in the brain is an independent predictor of clinical outcome; MLS(max) > 0.45 mm within 24 h or 2.35 mm within 48 h after intervention was associated with poor outcome at 90 days. When patients were grouped using the optimal MLS(max) thresholds, the groups with MLS(max) > 0.45 mm within 24 h after intervention and MLS(max) > 2.35 mm within 48 h after intervention had poorer prognosis, higher mortality, higher rates of decompression craniotomy, and lower improvements in mRS scores.

Patients generally undergo emergency endovascular treatment because important blood vessels are blocked or the infarct size is large. Under these circumstances, patients face the risk of reperfusion injury, vascular rupture/bleeding, and secondary cerebral edema; individual prognosis therefore varies greatly. The early prediction of disease outcome and disability severity, as well as the identification of high-risk patients, are crucial for patient treatment. Our findings suggest that MLS(max) > 0.45 mm within 24 h or > 2.35 mm within 48 h after intervention may be useful for alerting clinicians to provide early interventions, such as enhanced dehydration to reduce cranial pressure or early decompression craniotomy to improve patient outcomes. We also found that the median and threshold values of MLS(max) increased over time, which may be mainly caused by brain edema. Given that the average time of head CT reexamination within 24 h was 16 h and the average time of head CT reexamination within 48 h was 27 h, brain edema may begin to appear within 24 h after intervention in such patients.

Indicators including the core infarct site, lesion extent, degree of reperfusion injury, secondary cerebral hemorrhage, secondary cerebral edema, and midbrain aqueduct compression may affect the prognosis of patients with ACI, but the quantification of these indicators is complex and difficult to implement. By contrast, MLS in the brain can be easily quantified and its association with clinical deterioration has been well described^8^. We therefore explored the sensitivity and specificity of MLS for predicting outcomes in ACI patients. All patients included in our study had middle to large vascular lesions in the anterior circulation, and both the lesion scope and the affected functional area were relatively fixed. The mass effect caused by secondary cerebral hemorrhage and brain edema, which likely has a large effect on prognosis, was able to be expressed and quantified by MLS. To improve the accuracy of the study and represent intracranial lesions that may cause midline displacement, we selected four representative regions—the midbrain center, pineal calcification, septum pellucidum, and falx cerebri—to identify the optimal detection point for MLS, to facilitate its clinical implementation. Although the MLS values of all four sites had predictive value for the 90-day prognosis of patients, the MLS(max) tended to be a better neuroimaging predictor.

The MLS threshold as a predictor of poor prognosis has been widely studied in patients with stroke^8–15^. In a study of ischemic stroke, Pullicino et al. revealed that a threshold of horizontal pineal displacement > 4 mm on CT within 48 h of stroke onset can identify patients with a low probability of 14-day survival^17^. Subsequently, Haring et al. reported that MLS has high specificity (96.7%) but low sensitivity (19.4%) for predicting malignant middle cerebral artery infarction^18^. In another study, the presence of MLS, MLS > 1 cm, and infarct size were associated with 30-day mortality in a univariate analysis; however, logistic regression analysis revealed that infarct size but not MLS was an independent predictor of this outcome^19^. In a study published in 2012, MLS > 3.7 mm in follow-up CT approximately 24 h after severe ACI onset was able to predict a malignant course requiring surgical decompression^16^. Moreover, in a study by Wiggins et al., 40% of ICH patients with values above the threshold of MLS > 3 died^15^. Subsequent studies have reported that MLS > 6 mm within 48 h after ICH onset can predict 30-day mortality^11,12^, and that MLS > 6 mm within 24 h of symptom onset in symptomatic ICH patients is associated with poor prognosis^13,14^. Similarly, a recent investigation demonstrated that MLS(max) > 4 mm within 6 h after ICH may be a clinically significant threshold for poor prognosis^8^.

In the present study, the MLS(max) threshold of poor prognosis in patients with ACI after emergency endovascular treatment was 0.45 mm within 24 h after intervention, and 2.35 mm within 48 h after intervention. These are lower than the reported MLS thresholds for patients with ICH^8^. A possible reason for these discrepancies is that the study of ICH defined a good prognosis as mRS between 0 and 3, whereas we defined a good prognosis as mRS between 0 and 2, thus resulting in a smaller and more sensitive MLS threshold. However, it may also be that MLS within 24 h of ICH is mainly caused by hematoma expansion, and the MLS component caused by brain edema secondary to brain tissue necrosis is relatively low. Conversely, the main cause of midline displacement in patients with ACI may be the space-occupying effect of cerebral edema secondary to brain cell dysfunction, which may be combined with the expansion effect caused by secondary cerebral hemorrhage. Furthermore, a shift in the midline indicates that the brain cells themselves have been severely damaged and are necrotic. For patients with ACI, the MLS threshold that indicates poor prognosis is therefore relatively low.

In the current study, factors that may affect prognosis, such as age, preoperative NIHSS score, TOAST (Trial Org 10172 classification in Acute Stroke Treatment) classification, intravenous thrombolysis, time from onset to operation, operation time, left cerebral infarction, and postoperative bleeding, did not differ between the good and poor prognosis groups. This may be because, except for some patients with ACI within 6 h of onset who underwent direct endovascular intervention, all enrolled patients underwent preoperative CT perfusion imaging examination to assess the ischemic penumbra, and only patients who met the surgical guidelines underwent emergency endovascular treatment. Preoperative screening therefore likely eliminated any influence of preoperative basic information on the clinical outcomes of patients. Thus, CT perfusion imaging data and postoperative-related indicators should be used to predict clinical outcomes in such patients, rather than using basic preoperative information. However, it cannot be completely ruled out that the sample size in the present study was too small to detect any true differences; it is therefore necessary to expand the sample size for further confirmation of our findings.

The present study also has some limitations. First, it was conducted at a single center with a relatively small number of patients; the results therefore need to be further confirmed. This study will continue and we hope it will also be validated by other research groups. Second, our study only ensured that cranial imaging examination occurred within 24 h after intervention, but failed to complete cranial imaging examination at 24–48 h or more after intervention. We were therefore unable to investigate the relationship between time and the development of MLS(max) values, and were also unable to determine the optimal time point with the best predictive value of MLS(max).

## Conclusions

For the first time, we report the association between midline cerebral displacement and poor prognosis in patients with ACI who received endovascular therapy within 24 h of onset. Although the MLS value at each studied level had predictive value for the 90-day prognosis of patients, the MLS(max) tended to be a better predictor of prognosis. For predicting poor outcome, the optimal threshold was MLS(max) > 0.45 mm within 24 h or > 2.35 mm within 48 h after intervention.

## Data Availability

The datasets used and/or analysed during the current study available from the corresponding author on reasonable request.

## Abbreviations

ACI: Acute cerebral infarction
MLS: Midline shift
MLS(max): Maximal MLS for all MLS locations
ICH: Intracerebral hemorrhage
mRS: Modified Rankin Scale
CT: Computed tomography
IQR: Interquartile range
CI: Confidence interval
OR: Odds ratio
